# Magnitude of low birth weight and maternal risk factors among women who delivered in Debre Tabor Hospital, Amhara Region, Ethiopia: a facility based cross-sectional study

**DOI:** 10.1186/s13052-019-0683-1

**Published:** 2019-07-19

**Authors:** Maru Mekie, Wubet Taklual

**Affiliations:** 1 College of Health Sciences, Department of Midwifery, Debre Tabor University, P.O. Box: 272, Debre Tabor, Ethiopia; 2 College of Health sciences, Department of Population Health, Debre Tabor University, Debre Tabor, Ethiopia

**Keywords:** Low birth weight, Magnitude, Maternal risk factors, Ethiopia

## Abstract

**Background:**

Survival of newborns and long term complications are highly correlated with birth weight. The low birth weight rate is an indicator of a public health problem that includes long-term maternal malnutrition, ill health, and poor health care at population level during pregnancy. On an individual basis, low birth weight is an important predictor of newborn health and survival. We aimed to assess the magnitude of low birth weight and its associated factors among women who delivered in Debre Tabor Hospital (DTH), Amhara, Ethiopia.

**Methods:**

Facility based cross sectional study was employed on 282 mothers who delivered in DTH from December 2018 to March 2019. Single population proportion formula was used to calculate sample size. Data entry was completed in a template prepared in EpiData version 3.1 and analyzed by Statistical Package for Social Sciences (SPSS) version 20. Descriptive statistics were performed to describe the characteristics of the study participants. Crude and adjusted odds ratio with 95% confidence interval were used to identify the significance of association. A *p* value of < 0.05 was used to decide the significance of the association.

**Result:**

Of a total 282 interviewed mothers who delivered in DTH 12.0% (95%, CI: (8.5, 15.2%)) delivered low birth weight baby. Place of residence (AOR = 0.32, 95%, CI: (0.12, 0.85)), gravidity (AOR = 0.17, 95%, CI: (0.03, 0.97)), status of pregnancy (AOR = 0.29, 95%, CI: (0.09, 0.92)), and hemoglobin levels (AOR = 9.82, 95%, CI: (1.83, 52.73)) were found to be the significant predictors of low birth weight in this study.

**Conclusion and recommendation:**

Place of residence, status of pregnancy, gravidity, and level of hemoglobin were found to be statistically significant with low birth weight. Women who lived in urban areas, who had planned pregnancy, and gravida of < 5 had lower risk of giving low birth weight baby. Whereas, women who had hemoglobin level of < 11 mg/dl were more likely to deliver low birth weight baby. Being a multi-factorial problem, integrated and holistic approach shall be followed to reduce the prevalence, morbidity and mortality related to low birth weight.

## Background

The low birth weight (LBW) rate is an indicator of a public health problem that includes long-term maternal malnutrition, ill health and poor health care at population level during pregnancy. On an individual basis, it is an important predictor of newborn health and survival [1, 2].

LBW is defined by the World Health Organization (WHO) as weight at birth of less than 2,500 g (5.5 pounds) irrespective of the gestational age. It can be resulted from preterm birth (birth before 37 completed weeks) or small for term gestation (intra uterine growth restriction) or preterm as well as small for gestational age [3, 4]. Epidemiologically, term LBW is mostly the problem of developing countries which is highly linked to low socioeconomic status, and poor health care during pregnancy. While, LBW related to preterm birth is the problem of developed countries and usually related to congenital anomaly [3].

Globally, an estimated 18 million babies are born each year with LBW of which more than half, 9.3 million of them are in South Asia,and 3.1 million are in Sub-Saharan Africa [4]. According to the Ethiopia Demographic and Health Survey (EDHS) 2016, the prevalence of LBW in Ethiopia based on women’s self-report is reported to be 26% ((16% very small, 10% smaller than average) [1]. There is high health related costs among families of LBW baby [5]. LBW is closely associated with fetal and neonatal mortality and morbidity, inhibited growth and cognitive development, and chronic diseases later in life [3]. Numerous factors affect the duration of gestation and fetal growth, which in turn affects the birthweight. Those factors which cause LBW are related to either the fetus, the mother, or the physical environment or combination of such factors [3, 6, 7].

LBW is a multifactorial problem. According to different studies; maternal age of < 20 years, maternal body mass index (BMI) < 18.5), pregnancy interval of < 2 years, gestational age of < 37 weeks, no formal education, and being unmarried were found to be the significant predictors of LBW [6, 7]. Another study in central Ethiopia indicated that women with no formal education, no nutritional counseling during pregnancy, and those unmarried had higher odds of giving birth of LBW baby.

The government of Ethiopia is striving to reduce neonatal mortality by setting targets through establishing intervention at facility and community levels such as Integrated Management of Newborn and Childhood Illness (IMNCI), Newborn Corner Initiative (NBC), Neonatal Intensive Care Unit (NICU) Initiative, and Integrated Community Case Management (ICCM) [8]. Despite such initiatives have been undertaken by the government of Ethiopia and support bodies, neonatal mortality is sustained to be a great challenge [1, 8].

Affordable, accessible, and appropriate health care services are significant for preventing and treating LBW. Avoiding preventable factors of LBW is important to reduce the magnitude of LBW. Whereas, fully integrated appropriate neonatal and post neonatal medical and nutritional care for pre-term and small for gestational age infants are vital strategies to prevent morbidities and mortalities related to non-avoidable risk factors of LBW [4]. Thus, this study aimed to assess the magnitude and maternal risk factors of LBW among women who delivered in DTH of Amhara National Regional State, Ethiopia.

## Methods

### Study design and setting

Facility based cross-sectional study was conducted among women who attend delivery services in DTH from December 2018 to March 2019. The hospital is located in Debre Tabor town, 50 km East of Bahir Dar (the capital of Amhara National Regional State). The hospital provides service for more than 2 million population. According to the 2018 report, the hospital provided delivery services for more than 3600 women.

### Source and study population

All women who gave birth in DTH were considered as source population in this study. Women who gave birth during the study period and who fulfil the inclusion criteria were the study population. Women who give birth of preterm baby, births with comorbidities such as twin delivery, post term, preterm, still birth, pregnancy complicated with diabetes mellitus and hypertension were excluded.

### Sample size determination and sampling procedure

A single population proportion formula was used to identify sample size at 95% confidence level, 80% power, and a margin of error of 5%. The proportion of LBW was taken to be 21.23% from a facility based study in Bahir Dar [9]. The final sample size after adding 10% non-response rate was 282. Systematic random sampling technique was used to select the study participants. By considering the annual delivery services of the hospital in the previous year, the estimated delivery services for 3 months were 900. Thus, K = 900/282 ≈ 3. Data were collected for 3 months using systematic sampling technique of every 3 delivery cases. The next participant was used for those selected participants who failed to fulfil the inclusion criteria.

### Data collection procedures and quality assurance

The data collection process was undertaken by using structured questionnaire adapted from EDHS [1]. The questionnaire was organized in English language and translated to Amharic then translated back to English to check consistency. The tool consists of variables related to socio-demographic variables, obstetrics and related complications. The data collection process was performed by two trained BSc midwives and supervised by one master of public health professional. Clients’ medical charts were reviewed to check comorbidities and obstetrics characteristics.

The quality of data was maintained by using structured and pretested questionnaire. 2 days training was given to data collectors and supervisor to familiarize with the data collection tool. Strict supervision and monitoring were performed during the data collection period by the principal investigator. Moreover, appropriate recoding was performed after data entry to ease analysis.

### Measurements and definition

In this study obstetrics complication was defined as those who faced obstetrics problems such as still birth, preterm birth, and obstetric hemorrhage in their previous pregnancies. LBW is defined as new born weighing < 2500 g measured within 1 h of birth [3]. In this study, we have included only term newborns weighing < 2500 g as a case excluding preterm births.

#### Data processing and analysis procedures

The data entry was performed in a template prepared in EpiData version 3.1 and exported to SPSS version 20 for cleaning and analysis. Descriptive statistics such as frequency, and percentage were used. Chi-square test was performed to identify factors associated with the outcome variable. Variables with *p* value of < 0.2 in the chi-square analysis were entered in the multivariable logistic regression model to identify the independent predictors of LBW. Crude and adjusted odds ratio were used to identify the strength and direction of the association at 95% confidence interval. A *p*-value of < 0.05 was used to decide significance of association.

### Ethical consideration

This study was ethically approved by the Research Ethics Committee (REC) of College of Health Science, Debre Tabor University. Letter of permission was obtained from the hospital after briefing the purposes of the study. Written consent was obtained before initiation of data collection. Confidentiality of the information was maintained.

## Result

### Socio demographic characteristics of the study participants

The mean ages of the study participants were 28.82 years with standard deviation of ±5.27 and 28.08 years with standard deviation of ±5.17 among women with LBW and women without LBW respectively. With regards to residence, more than half, 158 (56.0%) of the participants were rural residents. The majority, 271 (96.1%) of the study participants were Orthodox Christians. Concerning to marital status, the majority, 267 (94.7%) were married and the rest, 15 (5.3%) were unmarried. With regards to occupation 183 (64.9%), 45 (16.0%), and 38 (13.5%) were house wives, farmers, and merchants respectively (Table 1).

**Table 1:** Socio-demographic characteristics of the study participants at DTH, 2019

Variables	Frequency	Percent	Chi-square (X^2^)	*P* value
Age in years				
18–19	5	2.0		
20–24	69	27.8	3.14	0.535
25–29	79	31.9		
30–34	72	29.0		
> =35	23	9.3		
Residence				
Urban	124	44.0	4.81	0.028
Rural	158	56.0		
Religion				
Orthodox	271	96.1		
Muslims	8	2.8	0.42	0.812
Others	3	1.1		
Ethnicity				
Amhara	280	99.3	0.28	0.871
Others	2	0.7		
Marital status				
Single	15	5.3	0.02	0.88
Married	267	94.7		
Occupation				
House wife	183	64.9		
Gov’t employee	16	5.7	2.00	0.57
Farmer	45	16.0		
Merchant	38	13.5		

### Obstetrics characteristics of the study participants

More than 40 % of the study participants were primi-gravida, and 8 (2.8%) were found to be grand multipara (pregnant for ≥5 times). With regards to parity, 130 (46.1%) were nulliparous. Whereas, 93 (33.0%) and 58 (20.6%) were para 1 and 2–4 respectively. More than a 10th, 29 (10.3%) of pregnancies were found to be unplanned. With regards to iron supplementation, 254 ((90.1%) of mothers were supplemented with iron sulfate. More than 90 %, 256 (91.5%) of mothers had antenatal care (ANC) follow-up. With regards to numbers of ANC visits, only 119 (42.2%) of mothers had reached 4 or more ANC visits. In this study 12.0% (95%, CI: (8.5, 15.2%)) of the newborns were LBW (Table 2).

**Table 2:** Obstetrics characteristics of the study participants at DTH, 2019

Variables	Frequency	Percent	Chi-square	*P* value
Gravidity				
1	123	43.6		
2–4	151	53.5	5.03	0.081
≥ 5	8	2.8		
Parity				
0	130	46.1		
1	93	33.0	0.97	0.807
2–4	58	20.6		
≥ 5	1	0.4		
Pregnancy status				
Planned	253	89.7	20.41	0.000
Unplanned	29	10.3		
ANC follow-up				
No visit	24	8.5		
1–3 visits	139	49.3	13.37	0.001
≥ 4 visits	119	42.2		
Iron supplementation				
Yes	254	90.1	16.41	0.000
No	28	9.9		
Hemoglobin level				
< 11 mg/dl	11	3.9	28.72	0.000
≥ 11 mg/dl	271	96.1		
Weight of the baby				
< 2500 g	34	12.0		
≥ 2500 g	240	88.0	¥	¥
Sex of the neonate				
Female	125	44.3	8.52	0.004
Male	157	55.7		
Rh status				
Positive	270	95.7	5.35	0.021
Negative	12	4.3		
Obstetrics complications				
Yes	15	5.3	0.94	0.332
No	267	94.7		

NB: - ¥: not applicable

### Factors associated with low birth weight

Variables with *p* value of < 0.2 were included in the multivariable model to identify the independent predictors of LBW. The variable “antenatal care” is excluded in the final model due to multicollinearity. Variables such as residence (AOR = 0.32, 95%, CI: (0.12, 0.85)), gravidity (AOR = 0.17, 95%, CI: (0.03, 0.97)), status of pregnancy (being planned/unplanned pregnancy) (AOR = 0.29, 95%, CI: (0.09, 0.92)), and hemoglobin levels (AOR = 9.82, 95%, CI: (1.83, 52.73)) were found to be statistically significant in the multivariable logistic regression model (Table 3).

**Table 3:** Multiple logistic regression analysis of factors associated with Low birth weight in DTH, 2019

Variables	Normal birth weight N (%)	Low birth weight N (%)	COR (95%)	AOR (95%)
Residence				
Urban	115 (46.4)	9 (26.5)	0.42 (0.19, 0.93)^a^	0.32 (0.12, 0.85)^b^
Rural	133 (53.6)	25 (73.5)	1	1
Gravidity				
1	109 (44.0)	14 (41.2)	0.21 (0.05, 0.99)^a^	0.17 (0.03, 0.97) ^b^
2–4	134 (54.0)	17 (50.0)	0.21 (0.05, 0.97)^a^	0.19 (0.04, 1.02)
≥ 5	5 (2.0)	3 (8.8)	1	1
Status of pregnancy				
Planned	230 (92.7)	23 (67.6)	0.16 (0.07,0.39)^a^	0.29 (0.09, 0.92)^b^
Unplanned	18 (7.3)	11 (32.4)	1	1
Iron supplement				
Yes	230 (92.7)	24 (70.6)	0.19 (0.08, 0.45)^a^	0.79 (0.22, 2.88)
No	18 (7.3)	10 (29.4)	1	1
Hemoglobin level				
< 11 g/dl	4 (1.6)	7 (20.6)	15.82 (4.35,57.52)^a^	9.82 (1.83, 52.73)^b^
≥ 11 g/dl	244 (98.4)	27 (79.4)	1	1
Sex of the newborn				
Female	102 (41.1)	23 (67.6)	2.99 (1.40, 6.41)^a^	2.00 (0.86, 4.65)
Male	146 (58.9)	11 (32.4)	1	1
Rh status				
Positive	240 (96.8)	30 (88.2)	0.25 (0.07, 0.88)^a^	0.68 (0.12, 4.00)
Negative	8 (3.2)	4 (11.8	1	1

*AOR* Adjusted Odds Ratio, *COR* Crude Odds Ratio, *CI* Confidence Interval

Key: ^a^ significant in bivariable analysis; ^b^ significant in multivariable analysis; 1: reference

## Discussion

LBW is one of the main risk factor for perinatal mortality especially in least developed countries [1, 4]. This study aimed to assess the magnitude of LBW and maternal risk factors among deliveries in DTH which could be very helpful to design appropriate measures to counteract the problem. In our study, the prevalence of LBW was found to be 12.0% (95%, CI: (8.5, 15.2%)). The finding is consistent with a study conducted in Dangla primary hospital, Amhara Region, Ethiopia [10]. However, the finding of our study is lower than the finding of the EDHS 2016 which revealed 26% of the births to be LBW [1]. The variation might be related to difference in the study setting and measurement of LBW in which women’s self-report was taken in the EDHS study unlike ours which is the actual weight at birth. Disregard of gestational age in the definition of LBW in EDHS report could also be the reason for the discrepancy unlike ours in which only term pregnancies were considered.

The odds of giving LBW baby were found to be lower among urban residents compared with rural residents (AOR = 0.32, 95%, CI: (0.12, 0.85)). This might be related to difference in awareness about nutrition and health in which urban women had better knowledge related to nutrition and health than rural women [11]. Difference in economic conditions might also elucidate the difference in which most of rural people are poor compared with urban counterparts [12]. Improving the socioeconomic status of mothers and providing awareness about the significance of health care during pregnancy shall be stressed.

With regards to gravidity, women with first pregnancy had 83% reduced risk of giving birth of LBW baby compared with those who had 5 or more pregnancy (AOR = 0.17, 95%, CI: (0.03, 0.97)). Women with higher gravidity are more likely to experience LBW compared with lower gravidity counterparts due to malnutrition which is highly related to frequent pregnancy with short inter pregnancy interval [6]. Moreover, women with higher parity are less likely to have antenatal care follow-up and may not have information about the importance of additional nutrition during pregnancy. The finding is not consistent with previous studies which revealed LBW among low parities despite the difference is not significant among women with parity of 5 or more in some studies [13, 14]. The discrepancy might be related to difference in study setting and study design.

Unplanned pregnancy was found to be a significant risk factor for LBW. The odds of giving birth of LBW baby were found to be lower among women whose pregnancy was planned compared with their unplanned pregnancy counterparts (AOR = 0.29, 95%, CI: (0.09, 0.92)). This might be related to failure of the mothers to have appropriate ANC follow-up which could be related to either lack of awareness or low socioeconomic condition. The finding of this study is supported by previous studies [15, 16]. Hence, avoiding unintended pregnancy through modern family planning methods is very imperative to reduce the incidence of LBW and its complications.

Women who took iron supplementation during pregnancy were less likely to give birth of LBW baby in the bivariable analysis compared with counterparts (COR = 0.19, 95%, CI: (0.08, 0.45)) though the significance of the association does not persist after adjusting for confounding factors. The finding of our study is consistent with a study conducted in Zimbabwe which revealed increased birth weight among women who took iron supplementation compared with their counterparts [17]. Encouraging women to take iron and folic acid during pregnancy is crucial to reduce the risk of LBW apart from reducing maternal morbidity and mortality related to anemia.

With regards to hematologic status, hemoglobin level was found to be significantly associated with LBW. The odds of giving birth of LBW baby were found to be 10 times higher among women with hemoglobin level of < 11 mg/dl compared with counterparts (AOR = 9.82, 95%, CI: (1.83, 52.73)). The finding is consistent with previous studies [18, 19]. Due emphasis shall be given about nutritional counseling especially iron reach foods during pregnancy to prevent LBW and related complications.

The multivariable logistic regression analysis revealed that there is no significant association between sex of the new born and LBW (AOR = 2.00, 95%, CI: (0.86, 4.65)). The finding is supported by a study conducted in Uganda [20]. However, the finding of our study is not consistent with a study conducted in University of Gondar Hospital, Northern Ethiopia [21]. The difference might be related to variation in sample size and case definitions in which the study at University of Gondar has included preterm births in the definition of LBW in which in our cases we have included only term babies.

This study shall be viewed in consideration of the following limitations. First the study was cross sectional study in which recall bias is a problem. Second the study was conducted in one health facility with small sample size which might affect generalizability. Similarly, nutritional status and eating habits of the study participants were not assessed which might affect birth weight. However, efforts were made to minimize bias by collecting data based on immediate experience of mothers and the interview was supplemented by chart review which could be taken as the strength of the study. Moreover, apart from using clear inclusion and exclusion criteria to recruit the study participants, pretested structured questionnaire was used for the data collection process which increases the quality of the study.

## Conclusion and recommendation

The prevalence of LBW in this study is lower than the EDHS report. Place of residence, gravidity, status of pregnancy, and hemoglobin level were found to be the significant predictors of LBW. Women who lived in urban areas, who had planned pregnancy, and gravida of < 5 had lower risk of giving LBW baby. Whereas, women who had hemoglobin level of < 11 mg/dl were more likely to deliver LBW baby. Being a multi-factorial problem, integrated and holistic approach shall be followed to reduce the prevalence, morbidity and mortality related to LBW.

## Data Availability

The datasets used in this study are available from the corresponding author on reasonable request.

## Abbreviations

ANC: Antenatal Care
DTH: Debre Tabor Hospital
EDHS: Ethiopia demographic and health survey
LBW: Low Birth Weight
SPSS: Statistical Package for Social Sciences
